# Supplementary material for: Visualisation of dCas9 target search in vivo using an open-microscopy framework
Source: Nat Commun. 2019 Aug 7;10:3552. doi: 10.1038/s41467-019-11514-0 (PMC6685946; doi:10.1038/s41467-019-11514-0)
Supplement: Supplementary file 1 — Supplementary Information [file 41467_2019_11514_MOESM1_ESM.pdf]

Visualisation of dCas9 target search *in vivo* using an open-microscopy framework

Martens et al., 2019

Supplementary Information

Contents

Supplementary Figure 1: Effect of state transitions on diffusion coefficient histogram 2
Supplementary Figure 2: The open-source miCube single-particle microscope 3
Supplementary Figure 3: Typical drift experienced by the miCube 4
Supplementary Figure 4: Individual pNonTarget and pTarget distributions 5
Supplementary Figure 5: no-sgRNA distributions fitted with MC-DDA or the target-binding model 6
Supplementary Figure 6: Effect of dCas9 on pTarget copy number 7
Supplementary Figure 7: Outline of the pLAB-dCas9 vector 8
Supplementary Figure 8: pNonTarget and pTarget construction and verifiction 9
Supplementary Figure 9: Schematic representation of obtaining cellular dCas9 copy number from number of tracks. 10
Supplementary Figure 10: Technical drawing of miCube component: Main cube 11
Supplementary Figure 11: Technical drawing of miCube component: Top cover 1 12
Supplementary Figure 12: Technical drawing of miCube component: Top cover 2 13
Supplementary Figure 13: Technical drawing of miCube component: Tube lens holder 14
Supplementary Figure 14: Technical drawing of miCube component: Excitation connector 15
Supplementary Figure 15: Technical drawing of miCube component: Bracket clamp 16
Supplementary Figure 16: Technical drawing of miCube component: Right-angled bracket 17
Supplementary Figure 17: Technical drawing of miCube component: Astigmatism block 18
Supplementary Figure 18: Technical drawing of miCube component: Camera mount 19
Supplementary Note 1: DNA and amino acid sequences 20
Supplementary Table 1: Descriptive list of miCube components. 22
Supplementary Table 2: List of vectors 26
Supplementary Table 3: List of oligonucleotides 27
Supplementary Table 4: List of primers. 27
Supplementary Table 5: Adjustment of the 405 nm laser power during sptPALM experiments. 28

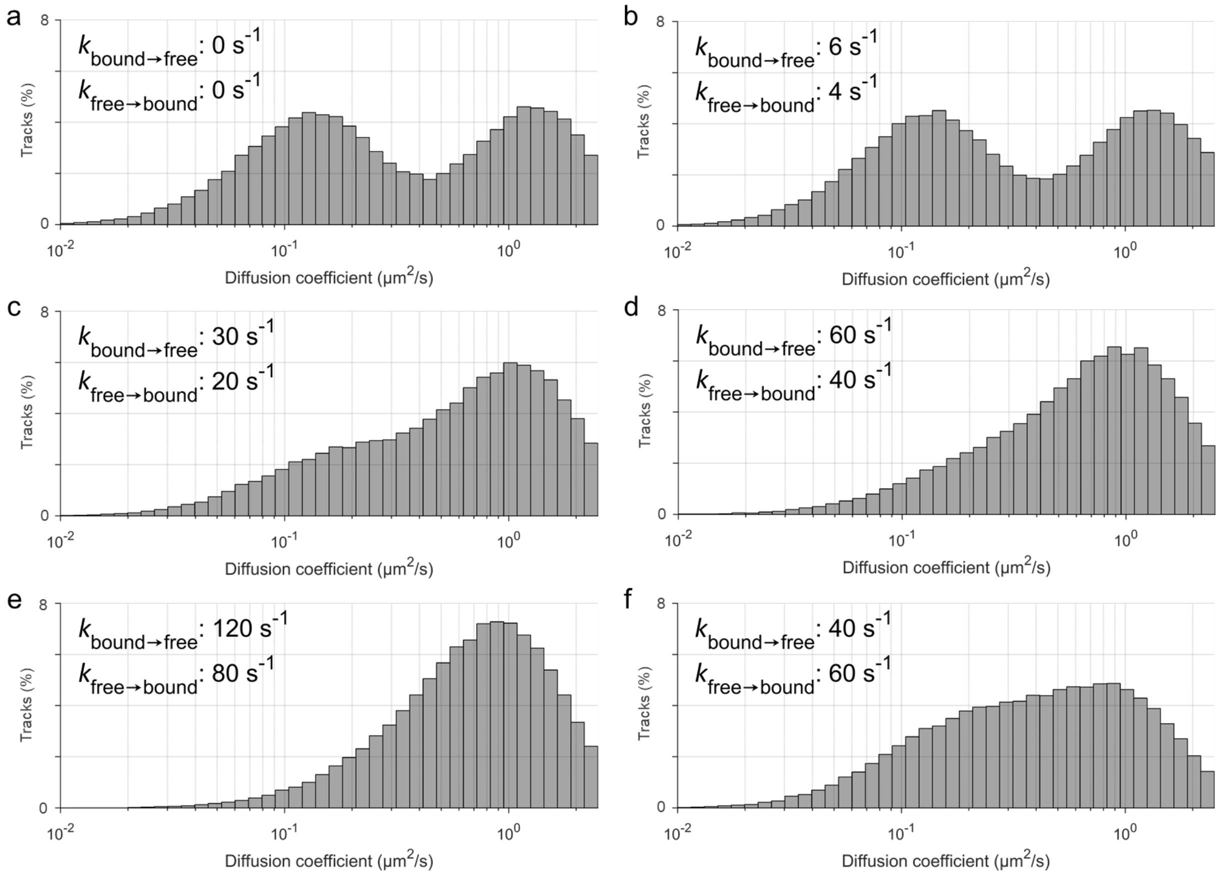

Supplementary Figure 1: Effect of state transitions on diffusion coefficient histogram

The pNonTarget model as described in Methods was ran with varying $k_{\text{bound→free}}$ and $k_{\text{free→bound}}$ values as indicated in the figure, while keeping the localization error and D_{free} constant at the values determined while fitting the actual data (38 nm and 2.0 μm^2/s, respectively). **a** Diffusion coefficient histogram if no state transitions would be present. **b** - **e** Diffusion coefficient histograms with the same $k_{\text{bound→free}}$: $k_{\text{free→bound}}$ ratio as the determined best-fitting values of ~ 3:2, while varying the absolute values of the two. **f** Diffusion coefficient histogram if the kinetic parameters were swapped.

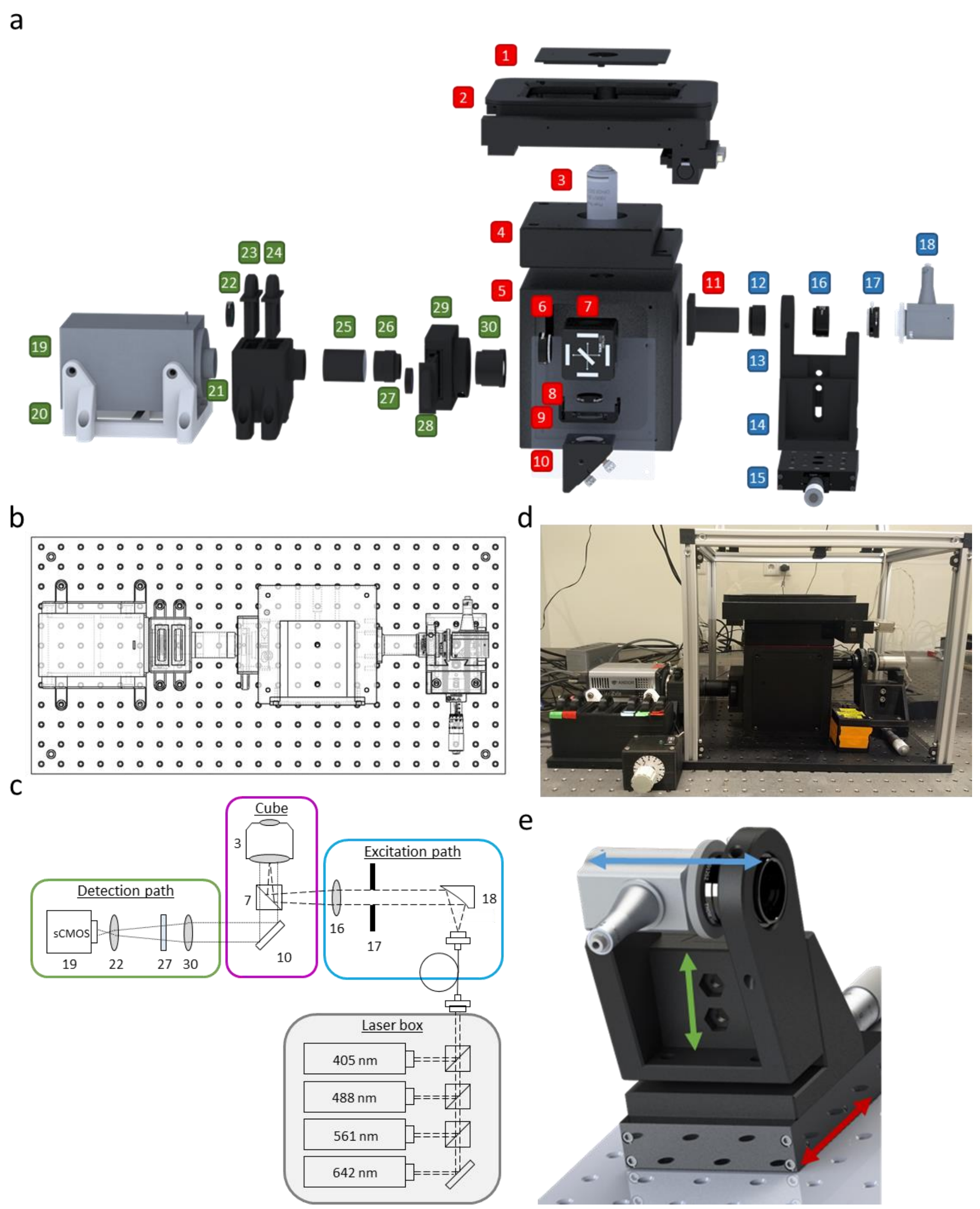

Supplementary Figure 2: The open-source miCube single-particle microscope

a Exploded render of the miCube highlighting individual components. A full list of components indicated by the numbered items can be found in Supplementary Table 5. **b** Top-down schematic view of the miCube on the breadboard, allowing clear view of mounting positions. Distance between mounting holes on the breadboard is 25 mm. **c** Schematic overview of the miCube instrument. Numbered items correspond to the items in **a** and Supplementary Table 5. The excitation path is visualized with dashed lines, the emission path is visualized with dotted lines. **d** Photograph of the fully assembled miCube as used for measurements in this manuscript. **e** Detailed view of the miCube excitation path. This sub-assembly is comprised of numbers 12-18. Arrows indicate isolated movement in the three spatial dimensions: distance from objective (blue), height of excitation unit (green), and horizontal position with respect to the objective (red).

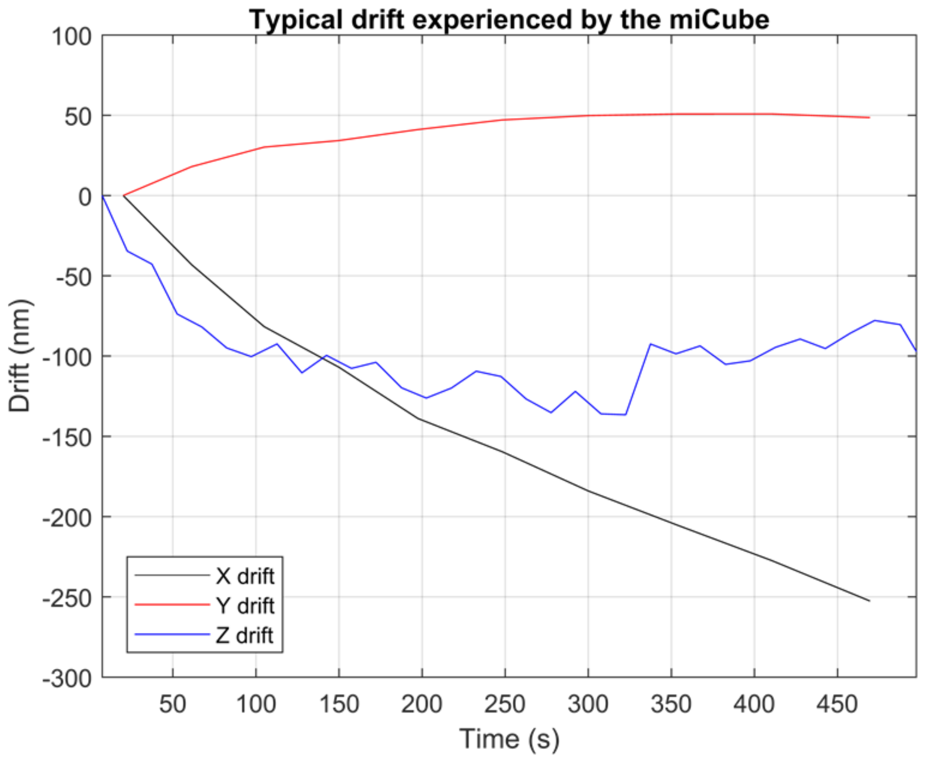

Supplementary Figure 3: Typical drift experienced by the miCube

Typical drift in X (black), Y (red), and Z (blue) as experienced by the miCube used throughout this study.

Repetition of this experiment led to the values specified in the main text.

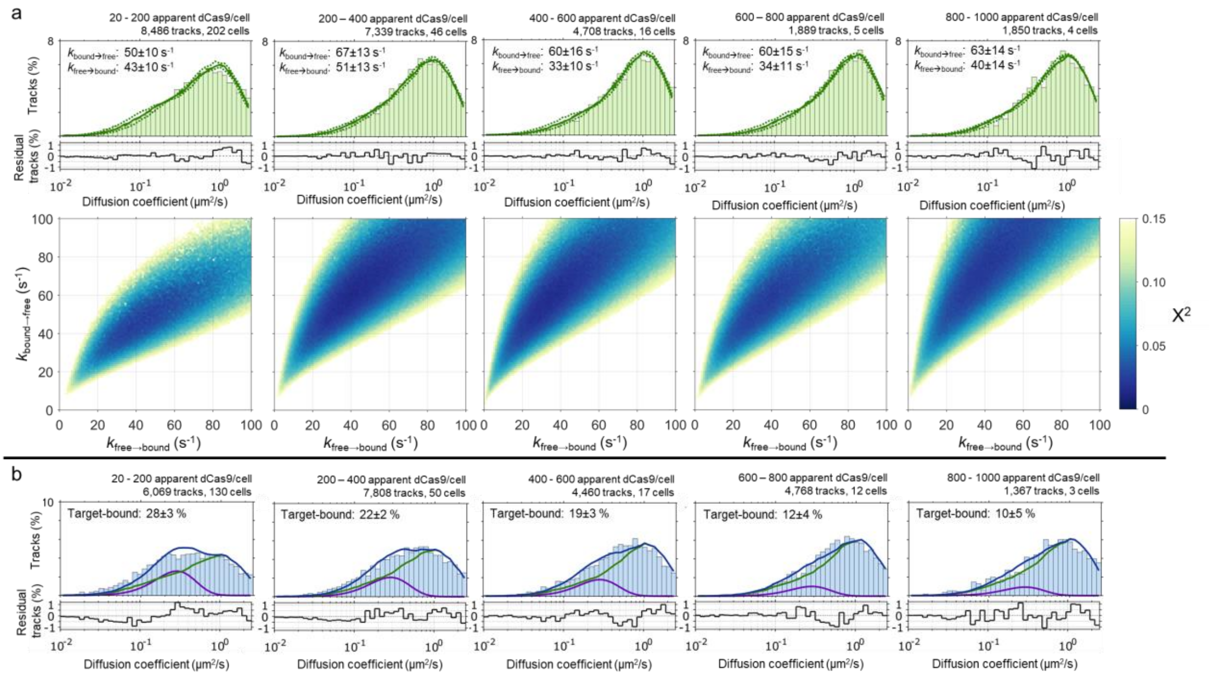

Supplementary Figure 4: Individual pNonTarget and pTarget distributions

a All five pNonTarget diffusional distributions fitted with MC-DDA, as explained in the main text, Methods section, and Fig. 2. At the bottom, the Chi-squared value is plotted for a range of MC-DDAs (100k simulated proteins) with different $k_{\text{free}\rightarrow\text{bound}}$ and $k_{\text{bound}\rightarrow\text{free}}$. **b** All five pTarget diffusional distributions fitted with the computational target-binding model, as explained in the main text, Methods section, and Fig. 3. Source data are provided as a Source Data file.

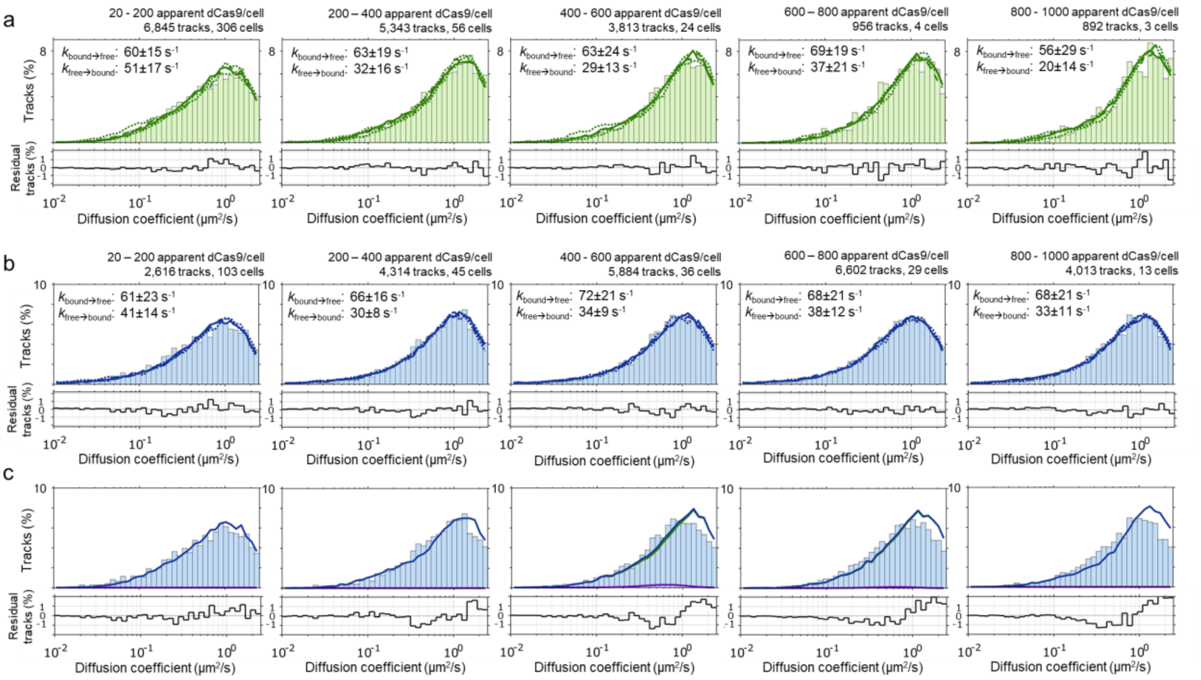

Supplementary Figure 5: no-sgRNA distributions fitted with MC-DDA or the target-binding model

a The five no-sgRNA pNonTarget diffusional distributions were fitted with MC-DDA as explained in the main text, Methods section. **b** The five no-sgRNA pTarget diffusional distributions were fitted with MC-DDA, as explained in the main text, Methods section. We note that the found kinetic rates are not significantly different from the no-sgRNA pNonTarget rates. **c** The five no-sgRNA pTarget diffusional distributions were fitted with a combination of the fixed no-sgRNA pNonTarget populations (from **a**) and a global non-fixed bound population. We note that no target-bound dCas9 population can be fitted, while the fitting methodology is identical to that of normal pTarget (Supplementary Figure 4 b). Source data are provided as a Source Data file.

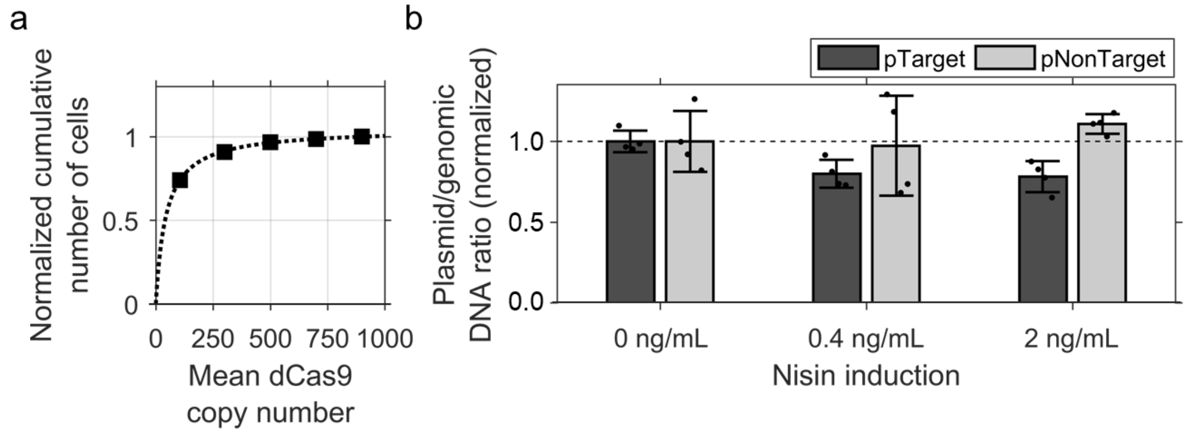

Supplementary Figure 6: Effect of dCas9 on pTarget copy number

a Representative normalized cumulative number of cells that have certain mean dCas9 copy numbers. Black squares are values taken from pNonTarget dataset, the dotted line is a fitted curve with equation y = 1.05 * [dCas9 copy number] / (44 * [dCas9 copy number]). **b** Normalized qPCR-determined ratio of plasmid:genome DNA for pTarget and pNonTarget for different Nisin induction. Error bars are the standard deviation determined from the average of two biological replicates (both averaged on two technical replicates). Individual data points are plotted as black circular markers. Source data are provided as a Source Data file.

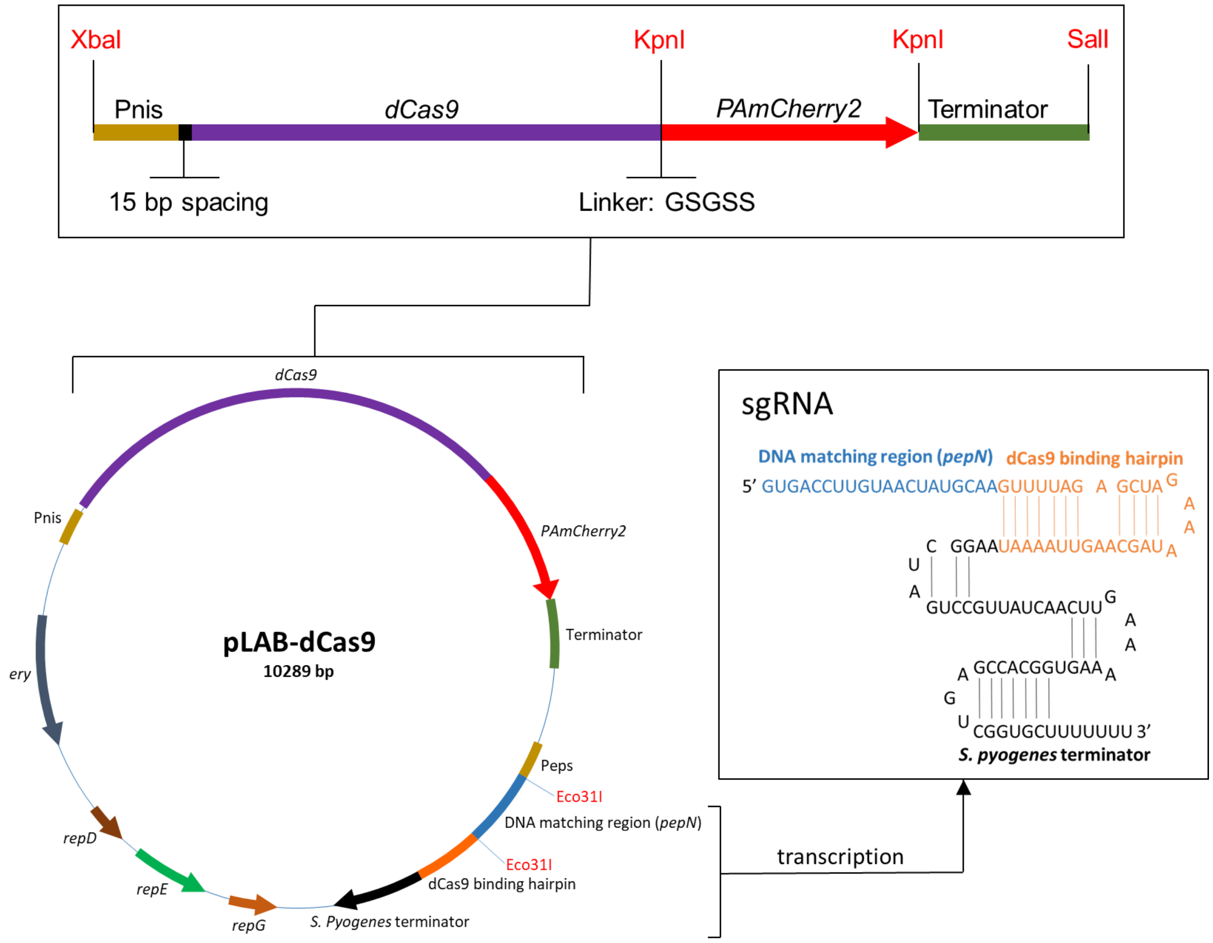

Supplementary Figure 7: Outline of the pLAB-dCas9 vector

Top: The sequence encoding dCas9[1] (*S. pyogenes*; AddGene.org plasmid #44249) is fused to the sequence encoding PAmCherry2[27] (AddGene.org plasmid #31932) with a flexible linker (amino acid sequence GSGSS), downstream of the *nisA*-promoter (Pnis) with an ribosomal binding site (15 bp spacing) and ending with a transcriptional terminator sequence derived from a lactococcal *pepN* gene. PAmCherry2 is flanked by two KpnI sites which should allow for interchanging fluorophores. The whole sequence is flanked by XbaI and SalI restriction sites to allow convenient cloning into a (expression) vector of choice. **Bottom:** The pLAB-dCas9 expression vector consists of PAmCherry2-labelled dCas9, an erythromycin resistance marker (Ery) and replication genes (*repD*, *repE* and *repG*)[73]. The *pepN* DNA matching region together with the dCas9 binding hairpin and the *S. pyogenes* terminator form the sgRNA, which is expressed under a constitutive promoter (Peps). Once the sgRNA molecule is transcribed, it folds to form the secondary structure that allows complex formation with dCas9.

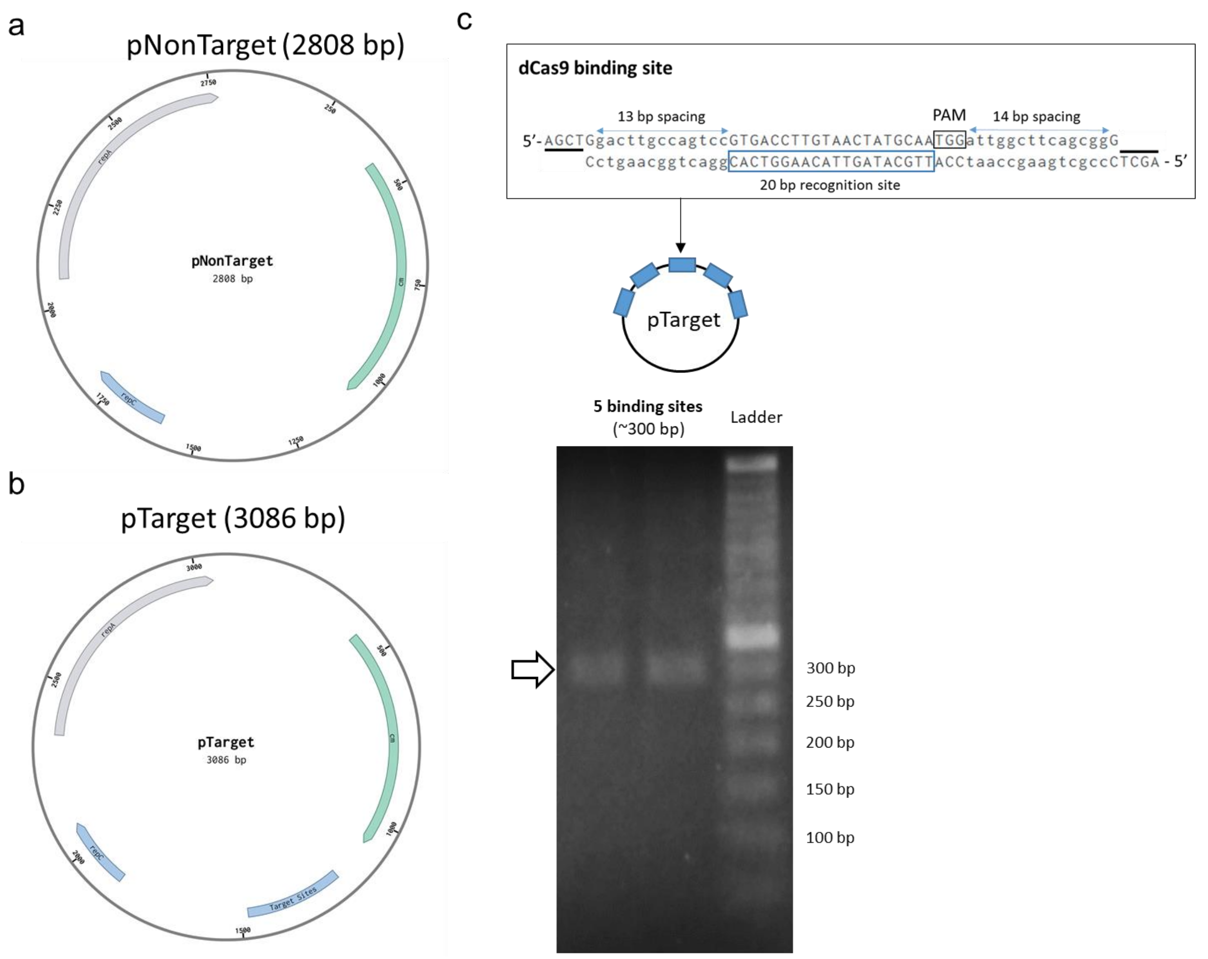

Supplementary Figure 8: pNonTarget and pTarget construction and verifiction

a,b Vector maps of pNonTarget and pTarget. Both targets contain *repA* and *repC* (DNA replication initiators) and a chloramphenicol-resistance marker (*cm*). Moreover, pTarget contains 5 target sites specified at 'Target Sites'. **c** dCas9 binding sites consisting of a 20 base pairs *pepN* recognition site, a 5'-NGG-3' PAM sequence, and spacing and overhang sequence motifs that are complementary to each other (indicated with black stripes) were annealed and ligated. This formed an array of five dCas9 binding sites in pNZ123, resulting in pTarget. Digestion and subsequent gel electrophoresis of plasmids isolated from two colonies revealed the expected length of the binding array in pTarget. One binding site is 54 base pairs in length, the final array of five binding sites is 278 base pairs (the expected PCR amplicon is 300 base pairs). Source data are provided as a Source Data file.

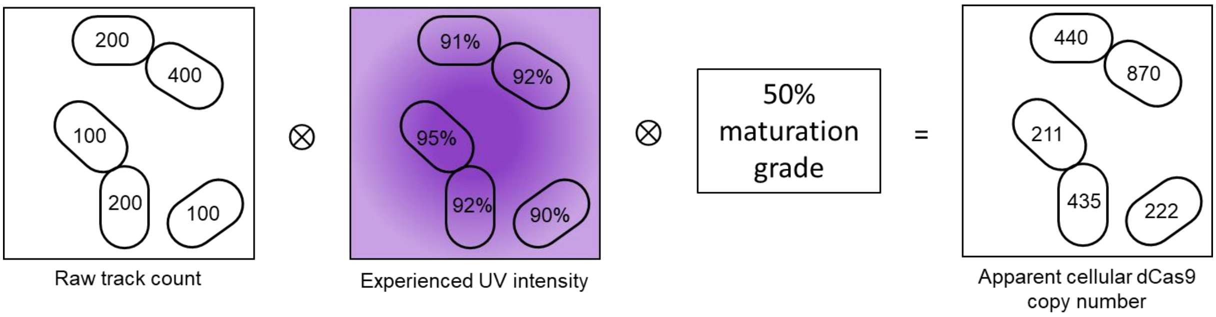

Supplementary Figure 9: Schematic representation of obtaining cellular dCas9 copy number from number of tracks

The raw track count (first subfigure) is convoluted with the experience UV intensity that the cell on average experienced (second subfigure; deduced via reflective scattering of excitation lasers), and with the expected maturation grade of PAmCherry2 (Methods).

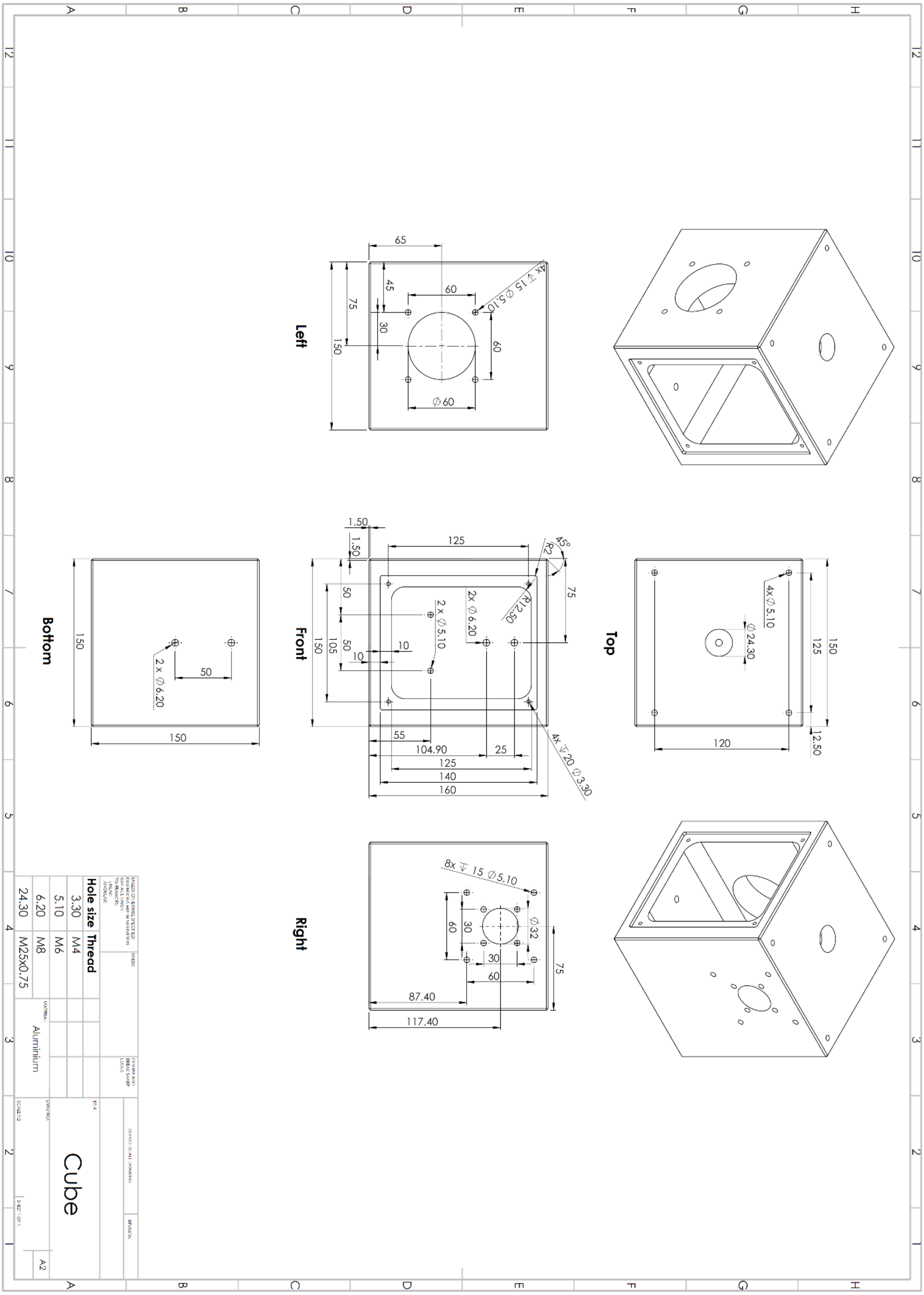

Supplementary Figure 10: Technical drawing of miCube component: Main cube

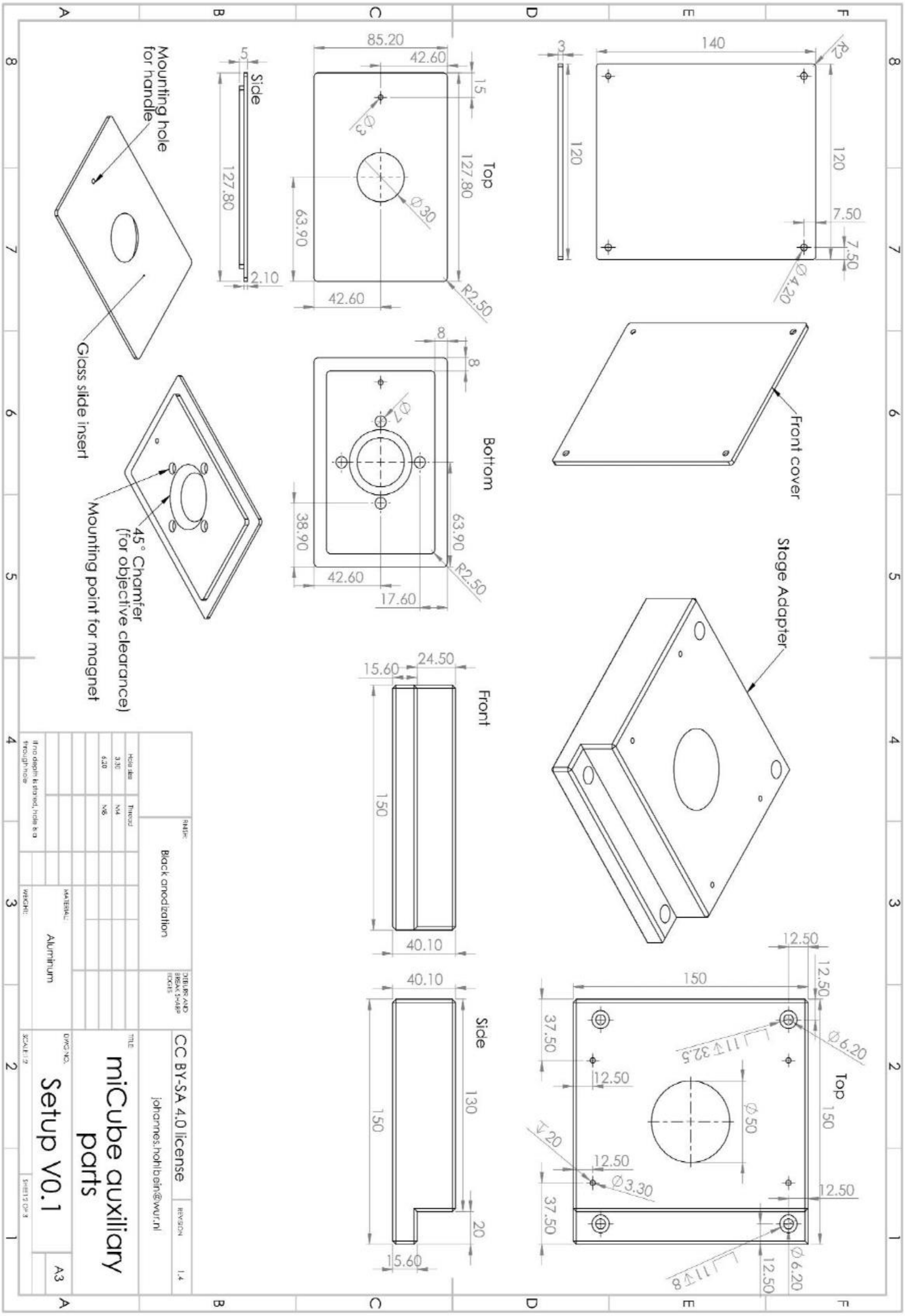

Supplementary Figure 11: Technical drawing of miCube component: Top cover 1

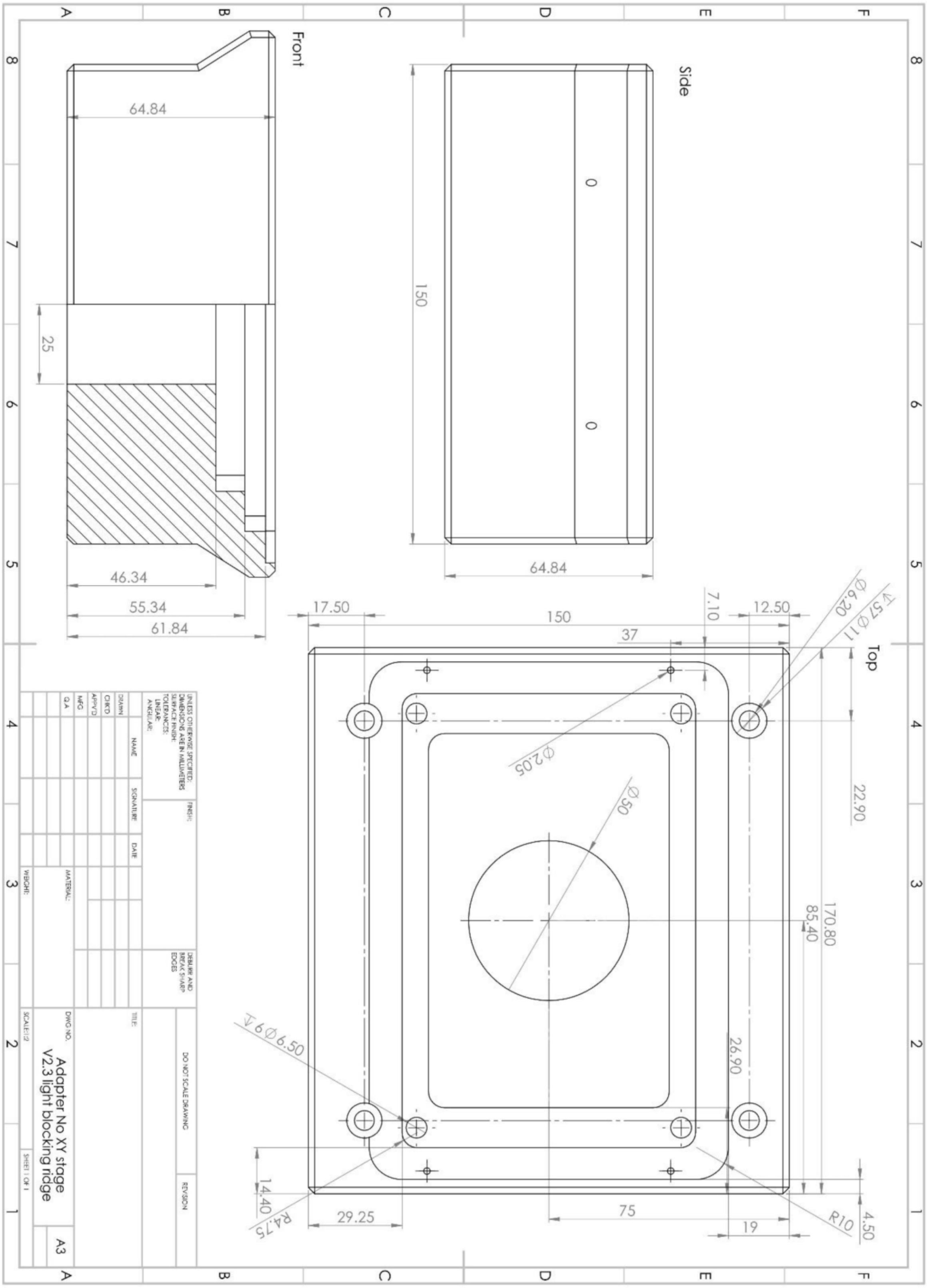

Supplementary Figure 12: Technical drawing of miCube component: Top cover 2

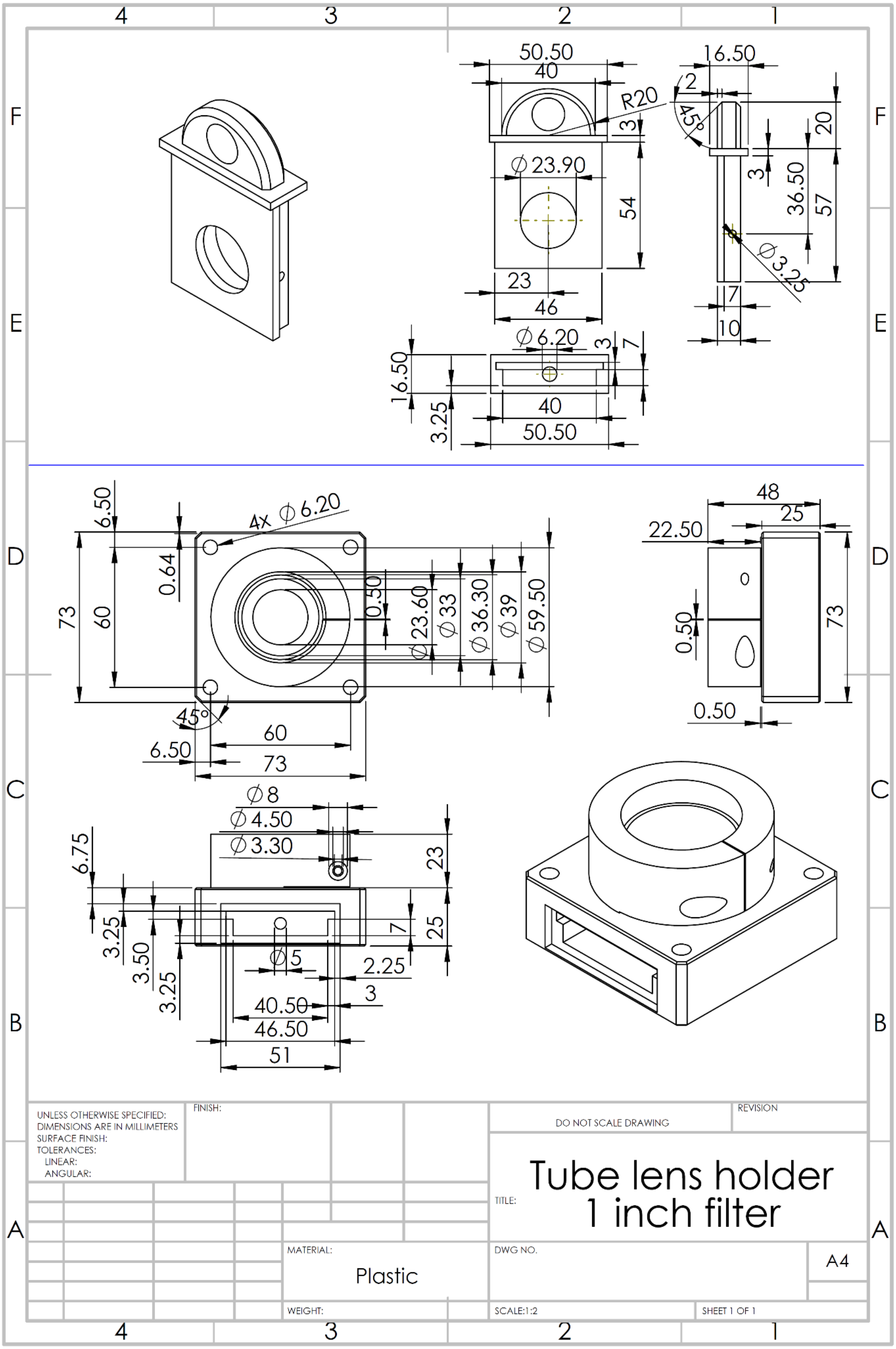

Supplementary Figure 13: Technical drawing of miCube component: Tube lens holder

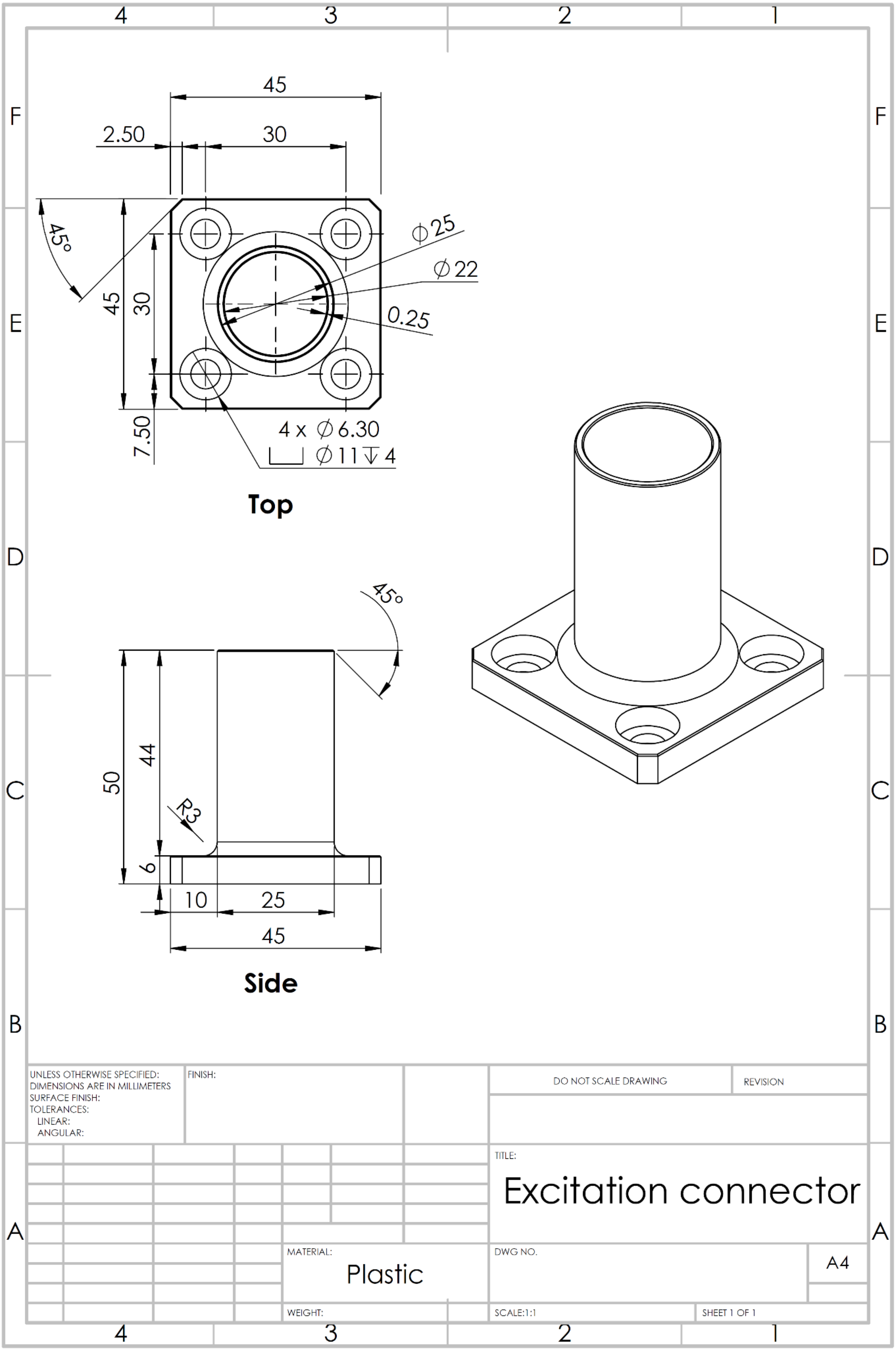

Supplementary Figure 14: Technical drawing of miCube component: Excitation connector

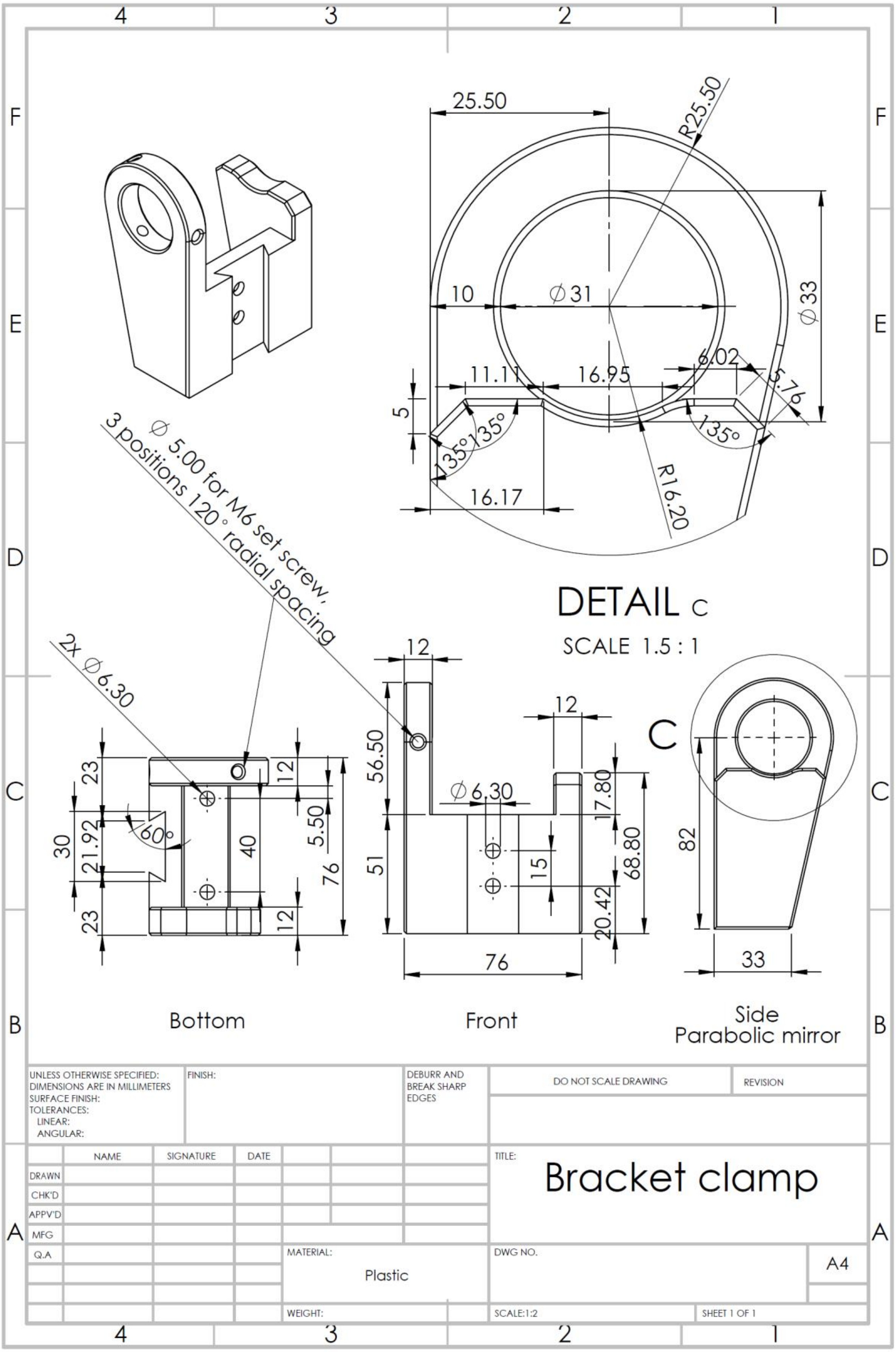

Supplementary Figure 15: Technical drawing of miCube component: Bracket clamp

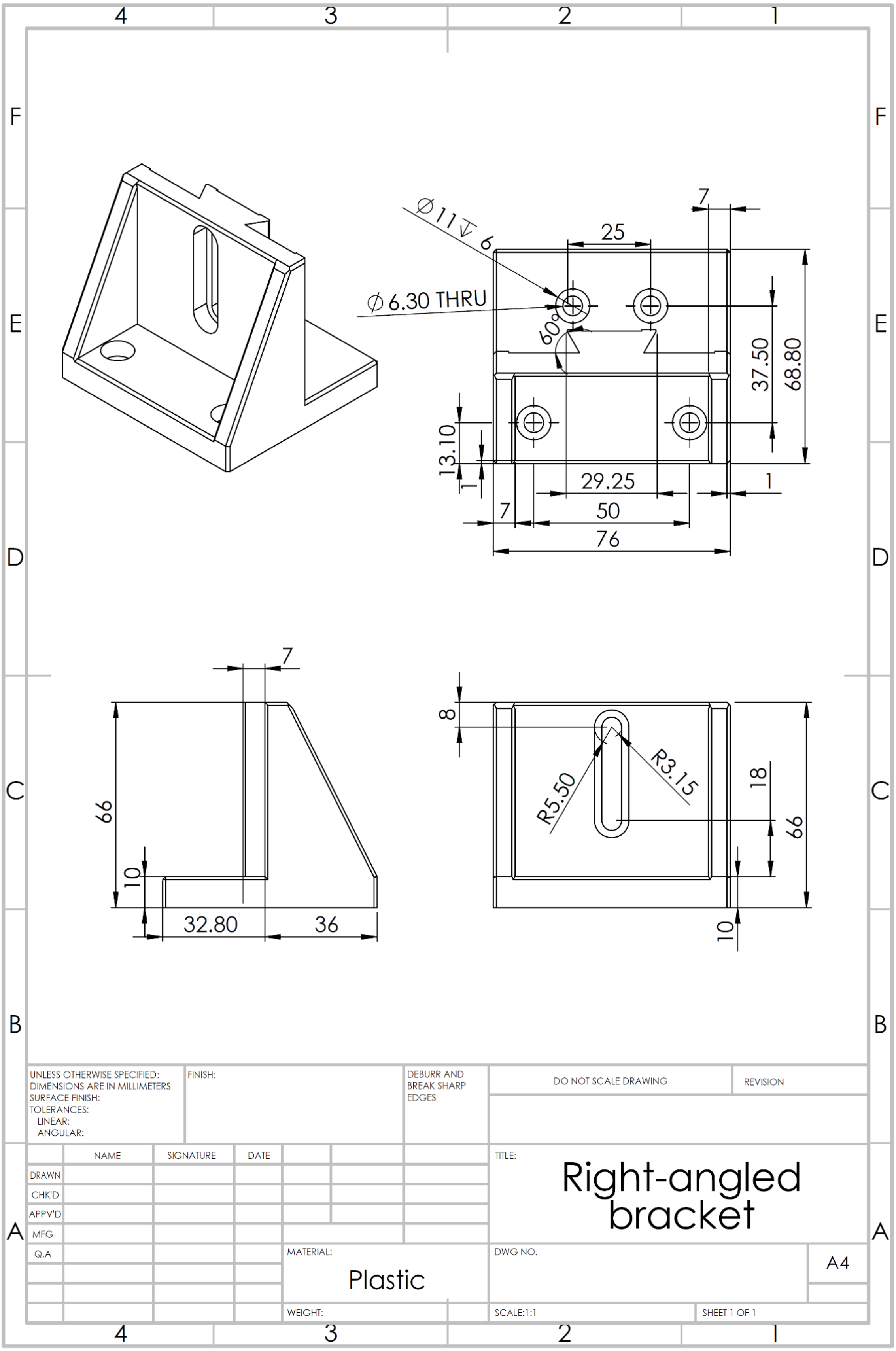

Supplementary Figure 16: Technical drawing of miCube component: Right-angled bracket

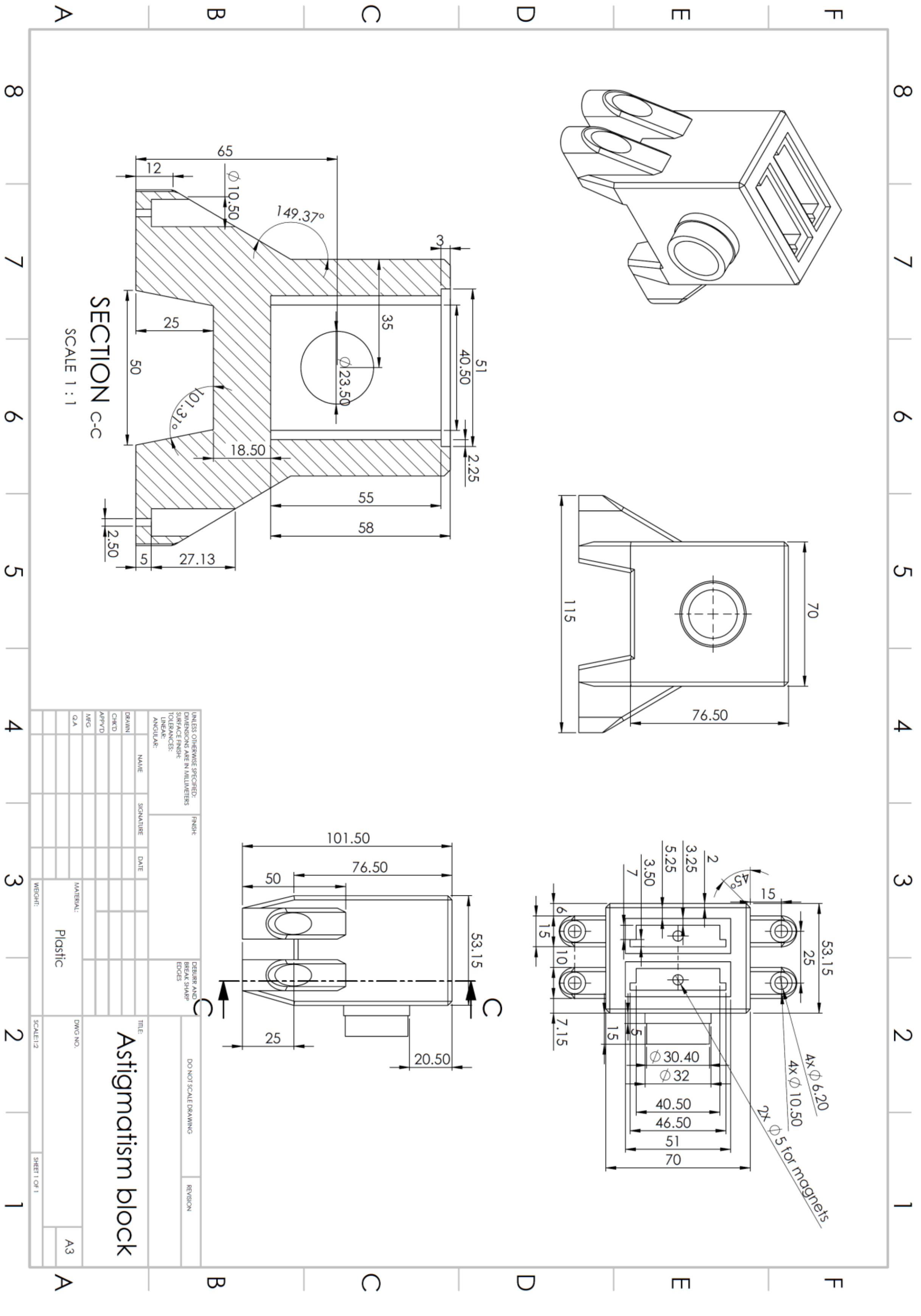

Supplementary Figure 17: Technical drawing of miCube component: Astigmatism block

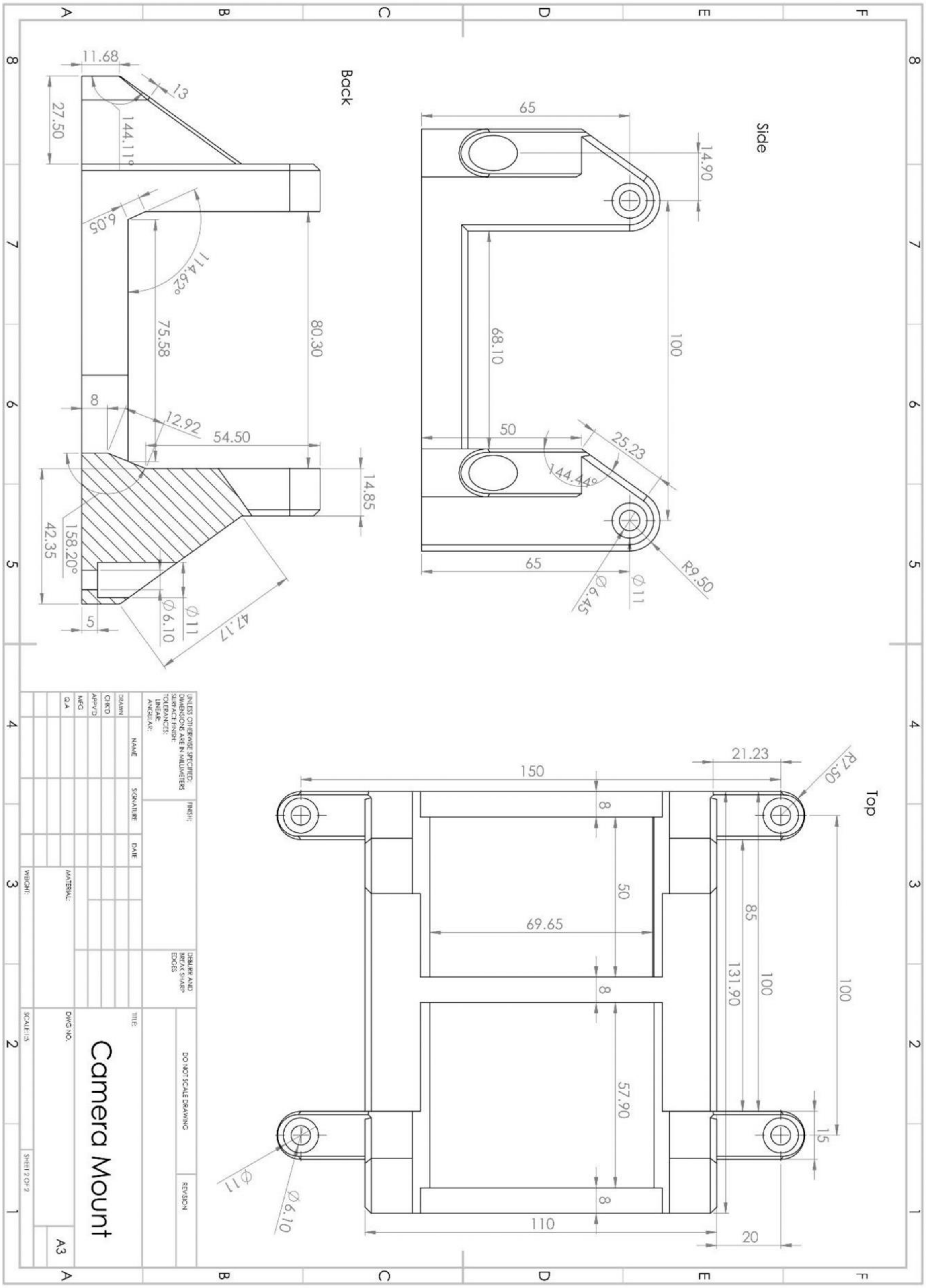

Supplementary Figure 18: Technical drawing of miCube component: Camera mount

Supplementary Note 1: DNA and amino acid sequences

> dCas9-PAmCherry2 DNA sequence

ATGGACAAAAAATACAGCATCGGCCTTGCCATCGGCACCAACAGCGTCGGGTGGGCCGTCATCACTGACGAGTATAAAGTGCCTAGTAAGAAGT
TCAAGGTGCTAGGCAACACTGACCGACACAGCATTAAGAAGAACCTGATCGGTGCTCTGCTATTCGATAGTGGTGAGACGGCCGAAGCTACAAG
ATTAAAAAGAACTGCAAGACGTAGATATACAAGACGTAAAAATCGTATTTGTTATTTACAGGAAATTTTTAGTAATGAGATGGCTAAGGTTGAT
GATAGTTTCTTTCATAGATTAGAAGAATCATTTTTAGTAGAAGAAGATAAAAAACATGAACGACATCCTATATTTGGAAATATAGTAGATGAAG
TAGCTTATCATGAAAAATATCCTACTATTTATCATTTACGTAAAAAATTAGTTGATAGTACTGATAAAGCTGATTTAAGATTGATATATTTAGC
ATTAGCACACATGATTAAATTTCGTGGTCATTTTCTAATTGAAGGAGATTTAAATCCTGATAACTCTGATGTTGATAAACTTTTTATTCAATTA
GTCCAAACTTATAATCAATTATTTGAAGAAAATCCAATTAATGCTAGCGGTGTAGATGCTAAAGCTATTTTATCAGCTAGATTAAGTAAAAGCA
GAAGACTAGAAAATTTAATTGCACAACTTCCTGGTGAGAAAAAGAATGGTTTATTTGGAAATTTGATTGCACTTAGTTTAGGTTTAACACCTAA
TTTTAAAAGTAATTTTGATTTAGCTGAAGATGCAAAACTTCAATTGAGTAAAGATACATATGATGATGATTTAGATAATTTATTAGCTCAAATT
GGTGATCAATATGCAGATTTATTTTTAGCTGCCAAAAATTTATCTGATGCTATTTTATTAAGTGATATATTACGTGTAAATACTGAAATTACTA
AAGCACCTTTATCAGCATCTATGATTAAAAGATATGATGAACATCATCAAGACTTAACATTATTAAAAGCATTAGTTAGACAACAATTACCAGA
AAAATATAAAGAAATTTTCTTTGATCAATCAAAAAATGGATATGCTGGATATATTGATGGTGGAGCATCACAAGAAGAATTTTATAAATTTATA
AAACCTATTTTAGAAAAAATGGATGGAACTGAAGAATTACTTGTTAAACTTAATAGAGAAGATTTATTAAGAAAACAAAGAACATTCGATAATG
GATCAATCCCACATCAAATTCATTTAGGTGAATTGCATGCTATTTTACGTAGACAAGAAGATTTTTATCCATTCTTGAAAGATAATAGAGAAAA
AATTGAAAAAATTTTAACTTTTAGAATTCCATATTATGTAGGACCTTTAGCACGAGGTAATTCTCGATTTGCATGGATGACACGTAAATCTGAA
GAAACAATTACACCATGGAATTTTGAAGAAGTTGTTGATAAAGGTGCTAGTGCACAATCTTTTATTGAAAGAATGACTAATTTTGATAAAAATT
TACCTAATGAAAAAGTATTACCAAAACATTCTTTATTATATGAATATTTTACTGTTTATAATGAACTTACAAAAGTAAAATATGTTACTGAAGG
AATGAGAAAACCAGCATTTTTATCAGGTGAACAAAAGAAAGCAATAGTTGATTTGTTATTTAAAACAAATCGTAAAGTTACTGTTAAACAACTT
AAAGAAGATTATTTTAAGAAAATTGAATGTTTTGATAGTGTTGAAATTTCTGGAGTTGAAGATAGATTTAATGCTAGTTTAGGTACATATCATG
ATTTATTAAAAATTATTAAAGATAAAGATTTTCTTGATAATGAAGAAAATGAAGATATTTTAGAAGATATTGTTTTAACATTAACATTATTTGA
AGATCGTGAAATGATTGAAGAACGTTTAAAAACATATGCACATTTATTTGATGATAAAGTAATGAAACAATTAAAAAGACGTAGATATACTGGA
TGGGGACGTTTATCTCGTAAATTAATTAATGGTATTAGAGATAAACAATCTGGTAAAACAATTTTAGACTTTCTAAAATCTGATGGATTCGCTA
ATCGTAATTTTATGCAATTAATTCATGATGATTCATTAACTTTTAAAGAAGATATTCAAAAAGCTCAGGTTAGTGGTCAAGGTGATAGCCTTCA
TGAACATATAGCTAACCTAGCTGGTAGTCCAGCAATTAAAAAAGGTATTTTGCAAACAGTGAAAGTAGTTGATGAACTTGTTAAAGTTATGGGT
CGTCATAAACCTGAAAACATTGTTATTGAAATGGCACGAGAAAATCAAACTACACAAAAAGGACAAAAGAATTCACGTGAACGTATGAAACGTA
TTGAAGAAGGTATTAAAGAACTAGGTAGTCAAATTCTTAAAGAACATCCAGTTGAAAATACACAATTACAAAATGAGAAATTATATTTATATTA
TTTACAAAATGGTCGTGATATGTATGTTGATCAAGAATTAGATATAAATCGCTTGTCAGATTATGATGTAGATGCAATTGTTCCTCAATCATTT
TTGAAAGATGATTCAATTGATAATAAAGTTTTGACACGTAGTGATAAAAATCGTGGTAAAAGTGATAATGTTCCTAGTGAAGAAGTTGTCAAGA
AAATGAAAAATTATTGGAGACAATTACTTAATGCTAAATTAATTACTCAACGTAAATTTGATAATTTAACAAAAGCAGAACGGGGAGGATTAAG
TGAACTTGATAAAGCTGGTTTTATAAAACGTCAATTAGTTGAAACAAGACAAATTACTAAACATGTAGCTCAAATATTAGATTCGCGTATGAAT
ACTAAATATGATGAAAATGATAAATTAATTAGAGAAGTTAAAGTTATAACATTAAAATCTAAATTAGTTAGTGATTTTAGAAAAGATTTTCAAT
TTTATAAAGTTCGTGAAATAAATAATTATCATCATGCTCATGATGCCTATCTTAATGCAGTAGTTGGAACAGCTTTAATTAAAAAATATCCAAA
ACTTGAAAGTGAATTTGTTTATGGTGATTATAAAGTCTATGATGTTCGCAAAATGATTGCTAAATCTGAACAAGAAATTGGTAAAGCTACAGCT
AAATATTTCTTTTATAGTAATATTATGAATTTCTTTAAAACTGAAATTACTTTAGCAAATGGAGAAATTAGAAAAAGACCATTAATTGAAACTA
ATGGTGAAACTGGAGAAATTGTTTGGGATAAAGGAAGAGACTTTGCAACAGTACGTAAAGTGTTATCTATGCCTCAAGTAAATATAGTTAAGAA
AACTGAAGTTCAAACAGGCGGATTTAGTAAAGAATCTATCTTACCAAAAAGAAATAGTGATAAATTAATTGCTCGTAAGAAAGATTGGGACCCT
AAAAAATATGGTGGTTTTGATTCTCCAACTGTCGCTTATTCGGTCTTAGTTGTTGCTAAAGTAGAAAAAGGTAAAAGTAAAAAATTAAAATCAG
TTAAAGAATTGTTAGGTATTACTATTATGGAAAGAAGTTCATTTGAAAAGAATCCTATTGACTTTTTAGAAGCCAAAGGTTACAAAGAGGTCAA
GAAAGACCTGATCATCAAACTGCCAAAGTACTCTCTCTTTGAATTAGAAAATGGACGTAAAAGAATGTTAGCATCTGCTGGTGAATTGCAAAAA
GGAAATGAATTAGCATTACCTAGTAAATATGTAAATTTCTTATACTTAGCTTCTCATTATGAAAAATTAAAAGGTTCACCGGAGGACAACGAGC
AGAAGCAACTTTTCGTGGAGCAACATAAACACTACCTCGACGAGATAATCGAACAAATTAGCGAGTTTTCAAAACGCGTCATCCTTGCCGATGC
CAATTTAGATAAAGTTTTATCAGCTTATAATAAACATAGAGATAAACCTATTAGAGAACAAGCTGAAAATATTATTCATTTATTTACTTTAACT
AATTTAGGTGCACCAGCTGCATTTAAATATTTCGATACAACAATTGATCGAAAAAGATATACATCAACTAAAGAAGTTTTAGATGCAACATTAA
TACATCAATCAATTACAGGATTATATGAAACACGTATTGATTTATCTCAATTAGGTGGTGATGGATCCGGAAGTTCAGCTATTATTAAAGAATT
TATGCGTTTTAAAGTTCATTTAGAAGGTAGCGTTAATGGTCATGAATTTGAAATTGAAGGAGAAGGTGAAGGTAGACCATATGAAGGTACACAA
ACAGCTAAATTAAAAGTTACAAAAGGTGGTCCATTACCTTTTGCTTGGGATATTTTGTCACCACAATTTATGTATGGTTCAAATGCTTATGTTA
AACATCCAGCTGATATTCCAGATTATTTTAAATTATCATTTCCAGAGGGTTTTAAATGGGAAAGAGTTATGAATTTTGAAGATGGTGGTGTTGT
AACAGTTACACAAGATTCATCTTTACAAGATGGTGAATTTATTTATAAAGTTAAATTAAGAGGTACTAATTTCCCTAGTGACGGACCGGTGATG
CAAAAGAAAACCATGGGATGGGAGACATTAAGTGAACGTATGTATCCTGAAGATGGTGCGTTGAAAGGTGAGCTGAAAGCTAGAACTAAATTGA
AAGATGGAGGCCACTATGATACTGAAGTAAAAACAACGTATAAGGCTAAGAAACCCGTTCAGTTACCAGGGGCATATAACGTTAATCGTAAACT
AGACATAACCTCTCATAATGAGGATTACACGATAGTTGAGCAATATGAACGAGCTGAAGGGCTTCATAGCACAGGTGGAATGGATGAACTTTAT
AAAtaa

> dCas9-PAmCherry2 amino acid translation

MDKKYSIGLAIGTNSVGWAVITDEYKVPSKKFKVLGNTDRHSIKKNLIGALLFDSGETAEATRLKRTARRRYTRRKNRICYLQEIFSNEMAKVD
DSFFHRLEESFLVEEDKKHERHPIFGNIVDEVAYHEKYPTIYHLRKKLVDSTDKADLRLIYLALAHMIKFRGHFLIEGDLNPDNSDVDKLFIQL
VQTYNQLFEENPINASGVDAKAILSARLSKSRRLENLIAQLPGEKKNGLFGNLIALSLGLTPNFKSNFDLAEDAKLQLSKDTYDDDLDNLLAQI
GDQYADLFLAAKNLSDAILLSDILRVNTEITKAPLSASMIKRYDEHHQDLTLLKALVRQQLPEKYKEIFFDQSKNGYAGYIDGGASQEEFYKFI
KPILEKMDGTEELLVKLNREDLLRKQRTFDNGSIPHQIHLGELHAILRRQEDFYPFLKDNREKIEKILTFRIPYYVGPLARGNSRFAWMTRKSE
ETITPWNFEEVVDKGASAQSFIERMTNFDKNLPNEKVLPKHSLLYEYFTVYNELTKVKYVTEGMRKPAFLSGEQKKAIVDLLFKTNRKVTVKQL
KEDYFKKIECFDSVEISGVEDRFNASLGTYHDLLKIIKDKDFLDNEENEDILEDIVLTLTLFEDREMIEERLKTYAHLFDDKVMKQLKRRRYTG
WGRLSRKLINGIRDKQSGKTILDFLKSDGFANRNFMQLIHDDSLTFKEDIQKAQVSGQGDSLHEHIANLAGSPAIKKGILQTVKVVDELVKVMG
RHKPENIVIEMARENQTTQKGQKNSRERMKRIEEGIKELGSQILKEHPVENTQLQNEKLYLYYLQNGRDMYVDQELDINRLSDYDVDAIVPQSF
LKDDSIDNKVLTRSDKNRGKSDNVPSEEVVKKMKNYWRQLLNAKLITQRKFDNLTKAERGGLSELDKAGFIKRQLVETRQITKHVAQILDSRMN
TKYDENDKLIREVKVITLKSKLVSDFRKDFQFYKVREINNYHHAHDAYLNAVVGTALIKKYPKLESEFVYGDYKVYDVRKMIAKSEQEIGKATA
KYFFYSNIMNFFKTEITLANGEIRKRPLIETNGETGEIVWDKGRDFATVRKVLSMPQVNIVKKTEVQTGGFSKESILPKRNSDKLIARKKDWDP
KKYGGFDSPTVAYSVLVVAKVEKGKSKKLKSVKELLGITIMERSSFEKNPIDFLEAKGYKEVKKDLIIKLPKYSLFELENGRKRMLASAGELQK
GNELALPSKYVNFLYLASHYEKLKGSPEDNEQKQLFVEQHKHYLDEIIEQISEFSKRVILADANLDKVLSAYNKHRDKPIREQAENIIHLFTLT
NLGAPAAFKYFDTTIDRKRYTSTKEVLDATLIHQSITGLYETRIDLSQLGGDGSGSSAIIKEFMRFKVHLEGSVNGHEFEIEGEGEGRPYEGTQ
TAKLKVTKGGPLPFAWDILSPQFMYGSNAYVKHPADIPDYFKLSFPEGFKWERVMNFEDGGVVTVTQDSSLQDGEFIYKVKLRGTNFPSDGPVM

QKKTMGWETLSERMYPEDGALKGELKARTKLKDGGHYDTEVKTTYKAKKPVQLPGAYNVNRKLDITSHNEDYTIVEQYERAEGLHSTGGMDELYK*

The asterisk (*) represents the stop codon ‘taa’.

Supplementary Table 1: Descriptive list of miCube components.

Main cube			
Nr	**Description**	**Details**	**Manufacturer**
1	Glass plate insert		Custom built – CNC milled
2	ASI XYZ stage	MS-2000 stage with Piezoconcept Z-insert	Applied Scientific Instrumentation, Eugene, OR, USA; and Piezoconcept, Lyon, France
3	Objective	TIRF 1.49NA HP SR objective	Nikon, Amsterdam, The Netherlands
4	TopCover		Custom built – CNC milled
5	miCube block		Custom built – CNC milled
6	Neutral density filter	NE60A-A	Thorlabs GmbH, Dachau/Munich, Germany
7	Dichroic mirror holder	DFM1/M	Thorlabs GmbH, Dachau/Munich, Germany
	Dichroic mirror	ZT405/488/561rpc-UF2 or ZT405/488/561/640rpc-UF2	Chroma, Bellows Falls, VT, USA
8	TIRF filter	ZET405/488/561m-TRF or ZET405/488/561/640m-TRF	Chroma, Bellows Falls, VT, USA
9	Connector	C4W-CC	Thorlabs GmbH, Dachau/Munich, Germany
10	45° elliptical mirror	KCB1E/M and BBE1-E02	Thorlabs GmbH, Dachau/Munich, Germany
11	Cover		Custom built – 3D printed

Excitation path			
Nr	**Description**	**Details**	**Manufacturer**
12	Spacer		Custom built – 3D printed
13	Reflective collimator holder		Custom built – 3D printed
14	Right-angle mounting plate		Custom built – 3D printed
15	25 mm Translation Stage	PT1/M	Thorlabs GmbH, Dachau/Munich, Germany
16	TIRF lens	AC254-200-A-ML	Thorlabs GmbH, Dachau/Munich, Germany
17	Aperture	SM1D12SZ	Thorlabs GmbH, Dachau/Munich, Germany
18	Reflective collimator	RC12APC-F01	Thorlabs GmbH, Dachau/Munich, Germany
-	Laser box	Lighthub 6 containing 4 lasers at 405 nm (60 mW), 488 nm (200 mW), 561 nm (500 mW), and 642 nm (2x 200 mW).	Omicron, Rodgau-Dudenhofen, Germany

Emission path			
Nr	**Description**	**Details**	**Manufacturer**
19	Camera	Zyla 4.2 PLUS	Andor, Belfast, Northern Ireland
20	Camera Mount		Custom built – 3D printed
21	Astigmatism block		Custom built – 3D printed
22	Astigmatism lens	LJ1516RM or LJ1144RM	Thorlabs GmbH, Dachau/Munich, Germany
23	Astigmatism lens holder		Custom built – 3D printed
24	Astigmatism lens holder		Custom built – 3D printed
25	Cover	SC600, cut to length	Thorlabs GmbH, Dachau/Munich, Germany
26	Connector		Custom built – 3D printed
27	Emission filter	ET525/50m or ET595/50m or ET700/75m	Chroma, Bellows Falls, VT, USA
28	Emission filter holder		Custom built – 3D printed
29	Tube lens holder		Custom built – 3D printed
30	Tube lens	ITL200	Thorlabs GmbH, Dachau/Munich, Germany

3D printing (Ultimaker 2+) settings							
Nr	**Material**	**Support**	**Adhesion**	**Layer height (µm)**	**Top / Bottom Thickness (mm)**	**Wall Thickness (mm)**	**Infill %**
13/14	PLA	Yes	Brim (5)	200	1.0	1.2	20
20/26	ABS	Yes	Brim (5)	200	0.8	0.8	20
21/23/24/28	PLA	Yes	Brim (5)	200	1.0	1.6	20

Numbers are in accordance with Supplementary Figure 1. Entities marked with custom-built have their complete technical drawings present in Supplementary Figures 10-18. A more exhaustive list can be found on https://HohlbeinLab.github.io/miCube/component_table.html.

Supplementary Table 2: List of vectors

Vector	Relevant properties	Size (kb)	Selection marker	Reference
pNZ123	Vector replicating in *L. lactis.*	2.8	Chloramphenicol	de Vos, 1987
pLABTarget	Encoding functional Cas9 expression system	10	Erythromycin	van der Els et al., 2018
pNonTarget	pNZ123 without binding sites	2.8	Chloramphenicol	This study
pTarget	pNZ123 containing five binding sites cognate to *pepN* sgRNA	3.1	Chloramphenicol	This study
pLAB-dCas9-nosgRNA	Cas9 module of pLABTarget replaced with Pnis – dCas9 – PAmCherry2	10.2	Erythromycin	This study
pLAB-dCas9	pLAB-dCas9-nosgRNA with added *pepN* sgRNA under constitutive promotor	10.3	Erythromycin	This study

Supplementary Table 3: List of oligonucleotides.

**Experiment**	**Construct**	**Oligonucleotide (5'- 3')**
pTarget construction	Forms *pepN* dCas9 binding site with complementary overhangs upon annealing.	AGCTGGACTTGCCAGTCCGTGACCTTGTAACTATGCAATG GATTGGCTTCAGCGG
		AGCTCCCGCTGAAGCCAATCCATTGCATAGTTACAAGGT CACGGACTGGCAAGTCC
pLAB-dCas9 construction	Forms *pepN* sgRNA upon annealing.	TGATGTGACCTTGTAACTATGCAA
		AAACTTGCATAGTTACAAGGTCAC

Supplementary Table 4: List of primers.

Experiment	Construct	Primer sequence (F=forward, R=reverse)
PCR insert validations and colony PCR	For pNZ123 (to check *pepN* binding site insertion)	F: TGAGATAATGCCGACTGTAC<br>R: CATTCAGTCATCGGCTTTCA
	For pLAB-dCas9 (to check *pepN* sgRNA insertion)	F: TGATGTGACCTTGTAACTATGCAA<br>R: TTGAAGAACCCGATTACATGG
qPCR	Q1, Q2: pTarget/pNonTarget	F: ACGAAAGTCGACGGCAATAGTT<br>R: CGTTTGTTGAACTAATGGGTGC
	Q3, Q4: Nested pNonTarget	F: GGGAGCGGAGTTTGGAATTT<br>R: ATAACCTAACTCTCCGTCGC
	Q5, Q6: ColonyCount	F: TCGATATGCACGTTGTCACC<br>R: CCCTCTCAGCTGCAATCTCT
	Q7, Q8: Nested qPCR Colony	F: GTGCTGAACCAGCGATTACA<br>R: TTGCTTTCACGTCAAGTTGG

Supplementary Table 5: Adjustment of the 405 nm laser power during sptPALM experiments.

TIME (S)	APPROXIMATE 405 NM LASER POWER FOR PHOTO ACTIVATION (µW/CM²)
**0**	2.7
**30**	2.8
**50**	3.2
**70**	3.8
**100**	4.7
**130**	6.2
**150**	8.9
**180**	13.7
**200**	20.1
**220**	27.6
**240**	36.2
**260**	46.1
**280**	62.1
**300**	78.1
**320**	83.8
**340**	97.0
**360**	113.9
**380**	126.3
**400**	146.2
**430**	194.4
**450**	260.7
**470**	320.9
**490**	383.6
**510**	455.4
**530**	508.4
**540**	619.0
