# Supplementary material for: Visualisation of dCas9 target search in vivo using an open-microscopy framework
Source: Nat Commun. 2019 Aug 7;10:3552. doi: 10.1038/s41467-019-11514-0 (PMC6685946; doi:10.1038/s41467-019-11514-0)
Supplement: Supplementary file 3 — Description of Additional Supplementary Files [file 41467_2019_11514_MOESM3_ESM.pdf]

Description of Additional Supplementary Files

File name: Supplementary Software 1

Description: The Supplementary Software contains the scripts used for data analysis and an accompanying flowchart.
