# Supplementary figures and images for: Visualisation of dCas9 target search in vivo using an open-microscopy framework
Source: Nat Commun. 2019 Aug 7;10:3552. doi: 10.1038/s41467-019-11514-0 (PMC6685946; doi:10.1038/s41467-019-11514-0)

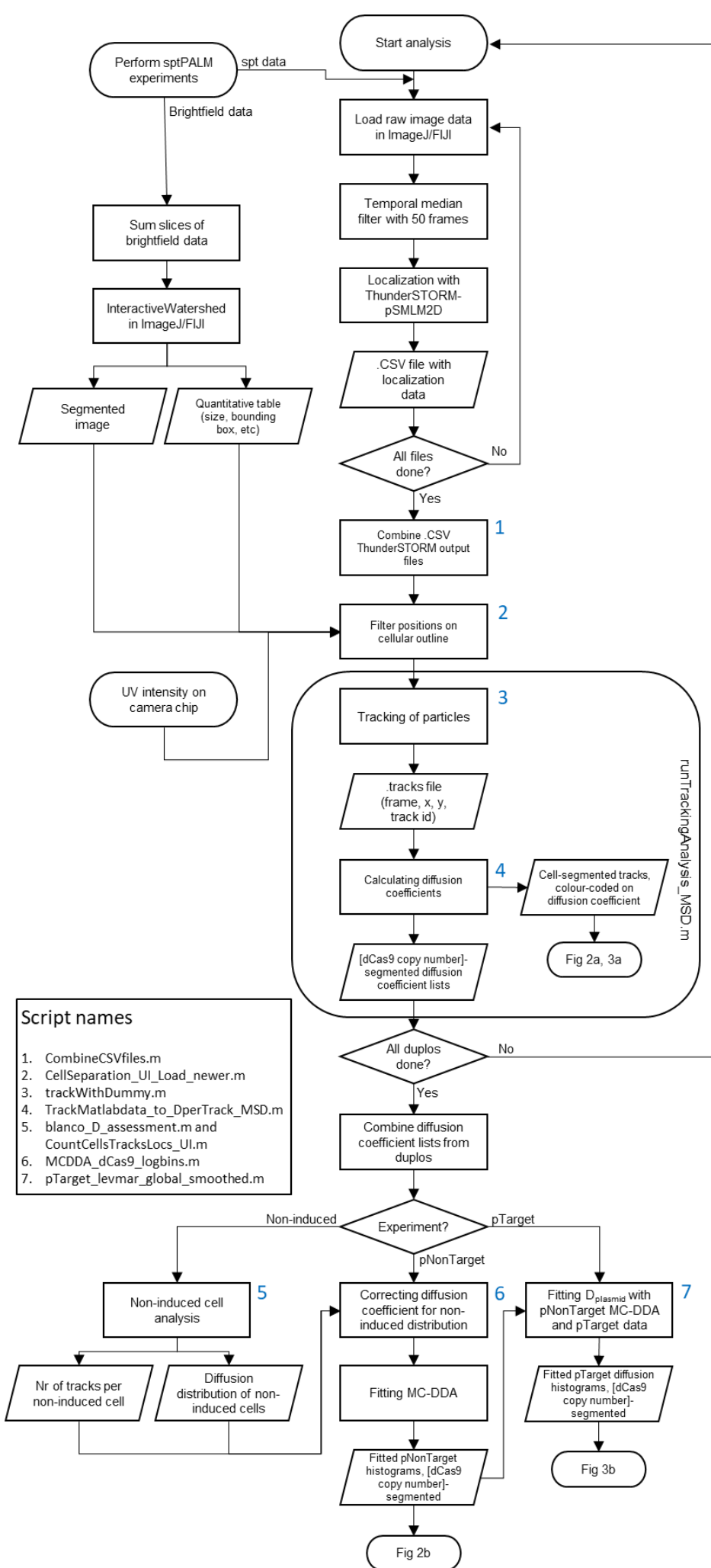

Supplement: Supplementary file 4 — Supplementary Software 1 [file 41467_2019_11514_MOESM4_ESM.zip › Scripts_NC_dCas9/Flowchart.pdf]
