# Supplementary material for: Visualisation of dCas9 target search in vivo using an open-microscopy framework
Source: Nat Commun. 2019 Aug 7;10:3552. doi: 10.1038/s41467-019-11514-0 (PMC6685946; doi:10.1038/s41467-019-11514-0)
Supplement: Supplementary file 5 — Reporting Summary [file 41467_2019_11514_MOESM5_ESM.pdf]

nature research

Corresponding author(s): Johannes Hohlbein

Reporting Summary

Nature Research wishes to improve the reproducibility of the work that we publish. This form provides structure for consistency and transparency in reporting. For further information on Nature Research policies, see Authors & Referees and the Editorial Policy Checklist.

Statistical parameters

When statistical analyses are reported, confirm that the following items are present in the relevant location (e.g. figure legend, table legend, main text, or Methods section).

n/a	Confirmed	
☐	☒	The exact sample size (*n*) for each experimental group/condition, given as a discrete number and unit of measurement
☐	☒	An indication of whether measurements were taken from distinct samples or whether the same sample was measured repeatedly
☒	☐	The statistical test(s) used AND whether they are one- or two-sided<br>*Only common tests should be described solely by name; describe more complex techniques in the Methods section.*
☒	☐	A description of all covariates tested
☐	☒	A description of any assumptions or corrections, such as tests of normality and adjustment for multiple comparisons
☐	☒	A full description of the statistics including central tendency (e.g. means) or other basic estimates (e.g. regression coefficient) AND variation (e.g. standard deviation) or associated estimates of uncertainty (e.g. confidence intervals)
☒	☐	For null hypothesis testing, the test statistic (e.g. *F*, *t*, *r*) with confidence intervals, effect sizes, degrees of freedom and *P* value noted<br>*Give P values as exact values whenever suitable.*
☒	☐	For Bayesian analysis, information on the choice of priors and Markov chain Monte Carlo settings
☒	☐	For hierarchical and complex designs, identification of the appropriate level for tests and full reporting of outcomes
☒	☐	Estimates of effect sizes (e.g. Cohen's *d*, Pearson's *r*), indicating how they were calculated
☐	☒	Clearly defined error bars<br>*State explicitly what error bars represent (e.g. SD, SE, CI)*

Our web collection on statistics for biologists may be useful.

Software and code

Policy information about availability of computer code

Data collection	Micromanager version 1.4.22
Data analysis	FIJI (ImageJ v. 1.51s) with plugins/scripts: ThunderSTORM-pSMLM version april_2018, Fast Temporal Median filter (https://github.com/marcelocordeiro/medianfilter-imagej), Interactive Watershed v 1.2.1.<br>MATLAB version 2017b and 2016b with custom scripts provided as supplementary data.

For manuscripts utilizing custom algorithms or software that are central to the research but not yet described in published literature, software must be made available to editors/reviewers upon request. We strongly encourage code deposition in a community repository (e.g. GitHub). See the Nature Research guidelines for submitting code & software for further information.

Data

Policy information about availability of data

All manuscripts must include a data availability statement. This statement should provide the following information, where applicable:

- Accession codes, unique identifiers, or web links for publicly available datasets
- A list of figures that have associated raw data
- A description of any restrictions on data availability

Sample raw data available, as well as all localization positional data, cell segmented data and diffusion coefficient lists available online at 10.5281/zenodo.1422461

Field-specific reporting

Please select the best fit for your research. If you are not sure, read the appropriate sections before making your selection.

☒ Life sciences ☐ Behavioural & social sciences ☐ Ecological, evolutionary & environmental sciences

For a reference copy of the document with all sections, see nature.com/authors/policies/ReportingSummary-flat.pdf

Life sciences study design

All studies must disclose on these points even when the disclosure is negative.

Sample size	For pNonTarget, we analysed 628 cells leading to 224.050 localisations forming 32.971 tracks of at least 4 localisations. For pTarget, we analysed 428 cells, 220.635 localisations, and 31.439 tracks. For the blanco, we analysed 110 cells leading to 5.353 localisations forming 803 tracks of at least 4 localisations. For no-sgRNA pNonTarget, we analysed 393 cells leading to 118.992 localisations forming 17.849 tracks of at least 4 localisations. For no-sgRNA pTarget, we analysed 226 cells leading to 172.798 localisations forming 23.429 tracks of at least 4 localisations.
Data exclusions	No data under the used experimental conditions was excluded
Replication	We replicated pNonTarget and pTarget measurement four times over the span of three days. We replicated no-sgRNA pNonTarget and pTarget measurements at least twice over the span of at least two days. The sample size specified above is the total of these replications.
Randomization	N/A
Blinding	No blinding was performed - this was unnecessary as all samples were treated identically

Reporting for specific materials, systems and methods

Materials & experimental systems

n/a	Involved in the study
☐	☒ Unique biological materials
☒	☐ Antibodies
☒	☐ Eukaryotic cell lines
☒	☐ Palaeontology
☒	☐ Animals and other organisms
☒	☐ Human research participants

Methods

n/a	Involved in the study
☒	☐ ChIP-seq
☒	☐ Flow cytometry
☒	☐ MRI-based neuroimaging

Unique biological materials

Policy information about availability of materials

Obtaining unique materials	A custom L. lactis strain was used. Full description on this strain is available in the supplementary materials and methods.
